# Endochin-like quinolone-300 and ELQ-316 inhibit *Babesia bovis*, *B. bigemina*, *B. caballi* and *Theileria equi*

**DOI:** 10.1186/s13071-020-04487-3

**Published:** 2020-12-03

**Authors:** Marta G. Silva, Reginaldo G. Bastos, J. Stone Doggett, Michael K. Riscoe, Sovitj Pou, Rolf Winter, Rozalia A. Dodean, Aaron Nilsen, Carlos E. Suarez

**Affiliations:** 1 Department of Veterinary Microbiology and Pathology, College of Veterinary Medicine, Washington State University, Pullman, WA USA; 2 Oregon Health and Science University, 3181 SW Sam Jackson Blvd., Portland, Oregon 97239 USA; 3 VA Portland Health Care System, 3710 SW US Veterans Hospital Road, Portland, OR 97239 USA; 4 Animal Disease Research Unit, Agricultural Research Service, USDA, WSU, Pullman, WA USA

**Keywords:** Bovine babesiosis, Equine piroplamsosis, *Babesia bovis*, *Babesia bigemina*, *Babesia caballi*, *Theileria equi*, Endochin-like quinolones, ELQ-300, nnELQ-316

## Abstract

**Background:**

The most common apicomplexan parasites causing bovine babesiosis are *Babesia bovis* and *B. bigemina*, while *B. caballi* and *Theileria equi* are responsible for equine piroplasmosis. Treatment and control of these diseases are usually achieved using potentially toxic chemotherapeutics, such as imidocarb diproprionate, but drug-resistant parasites are emerging, and alternative effective and safer drugs are needed. The endochin-like quinolones (ELQ)-300 and ELQ-316 have been proven to be safe and efficacious against related apicomplexans, such as *Plasmodium* spp., with ELQ-316 also being effective against *Babesia microti*, without showing toxicity in mammals.

**Methods:**

The inhibitory effects of ELQ-300 and ELQ-316 were assessed on the growth of cultured *B. bovis*, *B. bigemina*, *B. caballi* and *T. equi*. The percentage of parasitized erythrocytes was measured by flow cytometry, and the effect of the ELQ compounds on the viability of horse and bovine peripheral blood mononuclear cells (PBMC) was assessed by monitoring cell metabolic activity using a colorimetric assay.

**Results:**

We calculated the half maximal inhibitory concentration (IC_50_) at 72 h, which ranged from 0.04 to 0.37 nM for ELQ-300, and from 0.002 to 0.1 nM for ELQ-316 among all cultured parasites tested at 72 h. None of the parasites tested were able to replicate in cultures in the presence of ELQ-300 and ELQ-316 at the maximal inhibitory concentration (IC_100_), which ranged from 1.3 to 5.7 nM for ELQ-300 and from 1.0 to 6.0 nM for ELQ-316 at 72 h. Neither ELQ-300 nor ELQ-316 altered the viability of equine and bovine PBMC at their IC_100_ in* in vitro* testing.

**Conclusions:**

The compounds ELQ-300 and ELQ-316 showed significant inhibitory activity on the main parasites responsible for bovine babesiosis and equine piroplasmosis at doses that are tolerable to host cells. These ELQ drugs may be viable candidates for developing alternative protocols for the treatment of bovine babesiosis and equine piroplasmosis. 
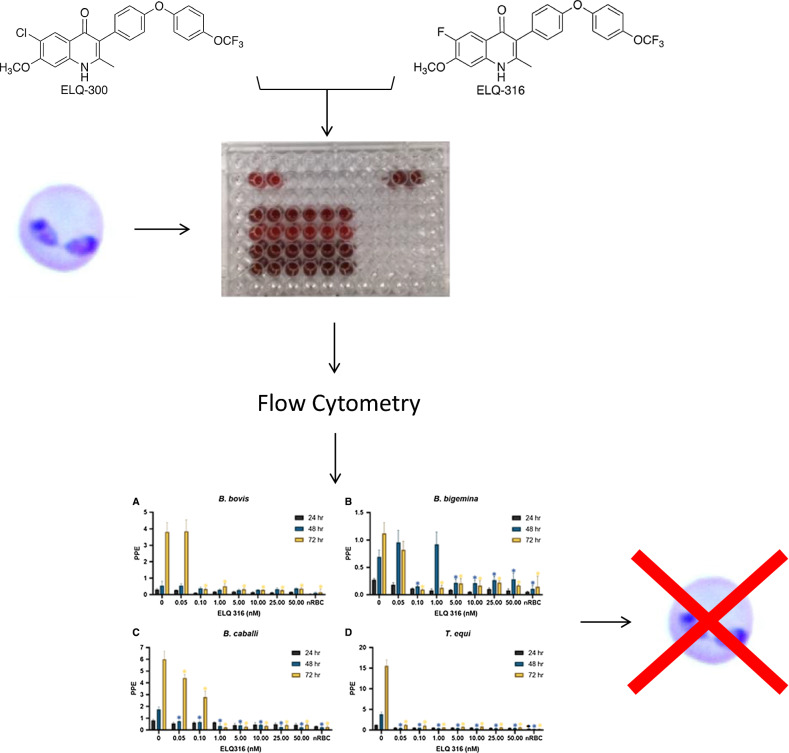

## Background

Tick-borne diseases caused by apicomplexan hemoparasites, such as those of genera *Babesia* and *Theileria*, impose serious economic impact on the cattle and horse industries worldwide [1, 2]. Babesiosis and theileriosis share similar acute disease signs, including anemia, loss of weight, anorexia and fever [3]. As a general rule, *Babesia* and *Theileria* are not eliminated in surviving animals and therefore can cause lifelong persistent infections. Important shared features among *Babesia* and *Theileria* species include a sexual reproductive cycle in their *Ixodes* arthropod hosts and asexual reproduction in the red blood cells (RBC) of their vertebrate hosts, a process that results in severe, potentially fatal hemolytic anemia [4–6].

*Theileria equi* and *Babesia caballi* are the etiological agents of equine piroplasmosis (EP), a disease that affects horses, mules, donkeys and zebras worldwide [7]. The threat of EP imposes severe and costly restrictions in the transportation of high-performance horses between endemic and non-endemic areas for participation in equestrian sporting events [3, 6]. No vaccines are currently available against *T. equi* and *B. caballi*, and considerable resources have been spent to develop drugs to treat animals against the harmful effects of acute EP and to prevent the loss of performance in chronically infected, high-value horses. Despite these efforts, horses that survive acute infection, especially when caused by *T. equi*, become persistently infected, asymptomatic carriers, a condition that can be associated with the resurgence of outbreaks of EP worldwide [8].

*Babesia bovis* and *B. bigemina* are two main causative agents of bovine babesiosis (BB), an acute and persistent economically important disease of cattle that typically causes high mortality [1]. While *B. bigemina* is usually associated with relatively milder acute hemolytic disease, *B. bovis* is implicated in a more severe presentation of the acute phase of the disease, characterized by the cytoadhesion of parasite-infected RBC in the brain capillaries, similar to that seen in cerebral malaria, and often leads to death [1].

Prevention and control of EP and BB have been typically achieved by controlling tick vector populations, the use of live attenuated vaccines in the case of BB and chemotherapy. The live, attenuated vaccines available to prevent acute BB, which are in use only in a limited number of countries, are only recommended for animals aged < 1 year and present several additional constraints, including the risk of reversion to virulence. Furthermore, cattle vaccinated with live, attenuated vaccines may also become persistently infected with the parasites and can serve as a reservoir for tick acquisition and transmission [9]. In addition, live vaccines can cause severe disease to immunocompromised and older cattle which may be more susceptible to the attenuated vaccine strains [9]. Given these scenarios, some animals vaccinated with live *Babesia* vaccines also need to be treated with anti-babesial drugs to prevent the development of acute disease caused by virulent escapes within the population of parasites in the attenuated vaccine strains. Currently, babesicidal drugs are the only option available for preventing losses due to babesiosis in adult vaccine-susceptible animals that need to be transported from non-endemic *Babesia* areas to endemic ones. Altogether, these aspects highlight the importance of having reliable babesicidal drugs to control the spread of outbreaks and prevent development of acute disease in herds vaccinated with live, attenuated *Babesia* vaccines.

Chemotherapy treatments based on diminazene aceturate and imidocarb dipropionate are the most effective and first-choice methods to manage animals with acute BB and EP [10, 11]. However, the efficacy of these drugs is highly variable, and treated animals need to be monitored closely for adverse effects, especially when high doses are used in attempts to achieve clearance of the parasites, which is a usual occurrence for valuable horses affected by EP [12]. In addition to toxic side effects, and although specific resistance to imidocarb by *Babesia* and *Theileria* parasites has not yet been documented, the potential for the development of drug resistance by *Babesia* parasites to other drugs, such as amicarbalide isethionate, has been previously recorded [13]. Consequently, there is the need to search for new effective and less toxic alternative chemotherapeutics against BB and EP.

Endochin-like quinolone (ELQ) compounds are potent selective inhibitors of the mitochondrial cytochrome* bc*_1_ complex, as demonstrated in *Plasmodium falciparum*, the causative agent of the most severe form of human malaria [14–17]. ELQ compounds have been shown to be highly effective against different species and multiple stages of *Plasmodium* [18, 19]. Importantly, the ELQ-300 and ELQ-316 compounds have been selected as pre-clinical anti-malaria candidates based on their reasonable oral bioavailability at efficacious doses, long half-life and metabolic stability [18, 19]. A recent study also demonstrated the efficacy of ELQ prodrugs combined with atovaquone to treat experimental babesiosis caused by *Babesia microti* in the immunodeficient mouse model [20]. Data from the study reported here showed that the combined therapy of ELQ and atovaquone resulted in complete clearance of the parasite, with no disease recrudescence even > 100 days after discontinuation of the treatment [20]. Based on these conclusive parasite inhibitory results, we evaluated the effect of ELQ-300 and ELQ-316 on the* in vitro* growth of *B. bovis*, *B. bigemina*, *B. caballi* and *T. equi*. Strong inhibition of the development of all these parasites, coupled with the lack of toxic effects on host cells, suggests that these two compounds are promising candidates for future development of novel alternative therapies to control BB and EP.

## Methods

### Synthesis of ELQ-300 and ELQ-316

The ELQ-300 and ELQ-316 compounds were synthesized as previously described [19, 21] (Additional file 1: Figure S1). Both compounds were kindly provided by the Department of Molecular Microbiology and Immunology, Oregon Health and Science University (Portland, OR, USA). Purity of both ELQ derivatives was assessed to be > 99% by proton-nuclear magnetic resonance spectroscopy and gas chromatography—mass spectrometry. ELQ-300 and ELQ-316 were diluted in 100% dimethyl sulfoxide (DMSO) to prepare stock solutions. Stock solutions were kept at room temperature until use. Working solutions were freshly prepared in parasite culture medium before being added to the parasite cultures.

### Cultures of* B. bovis*,* B. bigemina*,* B. caballi* and* T. equi*

*Babesia caballi* Puerto Rico strain [18], *B. bovis* Texas strain [19], *B. bigemina* Puerto Rico strain [20] and *T. equi* Florida strain [21] were grown in long-term microaerophilous stationary-phase cultures and incubated at 37 ℃ in an atmosphere of 5% CO_2_, 5% O_2_ and 90% N_2_, as previously described [22–25]. *Babesia bovis* and *B. bigemina* were grown in 96-well plates, in 180 µl per well of complete HL-1 culture media (pH 7.2; 2.38 g/l HEPES, 5 ml/l L-glutamine, 60 U/ml of penicillin G, 60 μg/ml of streptomycin and 0.15 μg/ml of amphotericin B; Sigma-Aldrich, St. Louis, MO, USA) supplemented with 40% bovine serum. Cultures contained a suspension of 10 and 5% packed cell volume of bovine erythrocytes for *B. bovis* and *B. bigemina*, respectively. *Babesia caballi* and *T. equi* were cultured under similar conditions, but the culture media were supplemented with 10 and 20% horse serum, respectively. In addition, *B. caballi* and *T. equi* cultures contained a suspension of 10% packed cell volume equine erythrocytes.

### Parasite growth inhibition assay

Growth inhibition assays using ELQ-300 or ELQ-316 were performed on cultured *B. bovi*s, *B. bigemina*, *B. caballi* and *T. equi* with a starting percentage of parasitized erythrocytes (PPE) of 0.2. Parasites were grown as described above in culture media containing different concentrations of ELQ-300 or ELQ-316 (range 0.05 to 50 nM) diluted in DMSO. Parasite cultures in the presence of DMSO (0.5 μl) and in the absence of the ELQ compounds were used as a positive control for parasite growth. Extra wells containing uninfected bovine or equine RBC were prepared and used as negative controls for the flow cytometric analysis. Fresh culture medium (150 µl/well) containing the respective drug concentration was replaced daily to parasite cultures. These experiments were carried out in triplicate for each tested concentration and controls, over a period of 72 h. PPE was monitored daily by flow cytometry, as previously described [22, 23]. The half maximal (50%) inhibitory concentration (IC_50_) values were calculated for ELQ-300 and ELQ-316 at 24, 48, and 72 h of incubation by extrapolation, with a 50% reduction of the PPE in the wells containing the ELQs compared with the positive control wells using nonlinear regression (GraphPad Prism ver. 8.0.2 for Windows; Graphpad Software Inc., San Diego, CA, USA). Similarly, maximal (100%) inhibitory concentration (IC_100_) values were also calculated at 72 h.

### Flow cytometric analysis for detection of parasite growth in cultures

The PPE of parasite cultures was determined by flow cytometry, as previously described [26, 27]. Briefly, 5 µl of the cultures was collected from the bottom of the wells and centrifuged at 450 *g* for 1 min at 4 ℃. The supernatant was discarded and the cell pelle washed twice with 150 μl of phosphate buffer saline (PBS) pH 7.2, following which the cell pellet was suspended in 200 μl of 25 μg/μl hydroethidine (HE) (Invitrogen, Carlsbad, CA, USA), incubated in 5% CO_2_ in an incubator at 37 ℃ for 20 min in the dark and ten washed twice with 200 μl of PBS to remove the excess HE. The supernatant was then discarded, and the cell pellet was suspended in 200 μl of fresh PBS. The suspended cells were analyzed by flow cytometry using a Guava® easyCyte flow cytometer (Luminex Corp., Austin, TX, USA) at a ratio of 800–1000 cells/µl with 20,000 events collected. The results were analyzed by FCS Express v6 (De Novo Software, Glendale, CA, USA). Normal, uninfected horse and cattle RBC were used as a negative control for the flow cytometric analysis.

### Effect of ELQ-300 and ELQ-316 IC_100_ on parasite growth

*In vitro* growth inhibition assays were performed over a period of 8 days using a starting PPE of 0.2 and 2%. Parasites were grown in the presence of calculated ELQ-300 or ELQ-316 IC_100_ values. Cultures growing in medium in the absence of the ELQs and non-infected RBC maintained in medium only were used as positive and negative controls, respectively. Culture medium containing the respective compound concentrations was replaced daily, 150 µl medium per well, for a period of 72 h, following which the parasites were cultivated in media only and split every 48 h for a period of 8 days. The PPE was evaluated at 24, 48, and 72 h, and 8 days of culture by flow cytometry.

### Cytotoxicity assay

Cytotoxicity of ELQ-300 and ELQ-316 in* ex vivo* peripheral blood mononuclear cells (PBMC) of bovine and equine were examined by exposing cells to the compounds at their calculated IC_50_ and IC_100_. For the bovine PBMC experiment, ELQ-300 IC_50_ and IC_100_ were 0.56 nM and 4.3 nM, respectively. And for the ELQ-316 IC_50_ and IC_100_ were 0.07 nM and 3.92 nM, respectively. For the equine PBMC experiment, ELQ-300 IC_50_ and IC_100_ were 0.23 and 5.94 nM, respectively, and ELQ-316 IC_50_ and IC_100_ were 0.11 and 6.18 nM, respectively. Viability of bovine and horse PBMC was evaluated by monitoring cell metabolic activity using a colorimetric assay. Briefly, peripheral blood was collected from healthy cattle and horses via jugular venipuncture into Vacutainer® tubes containing ACD (acid citrate dextrose) (Becton, Dickinson and Company, Franklin Lakes, NJ, USA) and PBMC were isolated using Histopaque® (Sigma-Aldrich) per standard protocol. Cells were then plated at 2 × 10^4^ cells/well in 96-well plates in complete Dulbecco’s modified essential medium (cDMEM; 10% fetal bovine serum, 24 mM of HEPES, 2 mM of l-glutamine, 100 IU/ml penicillin, and 100 ug/ml streptomycin) and incubated with the ELQ compounds. The Cell Proliferation WST-1 reagent (Roche Applied Science, Penzberg, Germany) was added to the cell cultures following the manufacturer’s protocol at 24, 48, and 72 h after exposure to the ELQ compounds. Absorbance at 440 nm was measured using an enzyme-linked immunosorbent assay (ELISA) plate reader at 4 h after adding the WST-1 reasgent to the cells. Cells in cDMEM in the absence of the ELQ compounds and cells exposed to DMSO only (1/400 dilution, which corresponds to the highest volume used on the diluted ELQs) were used as negative controls. PBMC exposed to concanavalin (Con) A diluted in cDMEM (5 μg/ml) (Sigma-Aldrich) and Draxxin® [22] were used as a positive control.

### Statistical analysis

Growth of parasites in culture was analyzed using one-way analysis of variance (GraphPad Prism version 8.0.2 for Windows; Graphpad Software Inc.). Values of *P* < 0.05 were considered to be statistically significant in terms of the effect of the ELQs on the parasite growth. Significant differences in PBMC viability were measured by Student’s t-test, and *P* values < 0.05 were considered to be significant.

### Ethical Statement

The* in vitro* cultures of *B. caballi*, *T. equi*, *B. bovis* and *B. bigemina* require erythrocyte and serum equine and cattle donors. The protocols used for the bleeding of the horse and cattle donors for were approved by the Institutional Animal Care and Use Committee (IACUC) of the University of Idaho (Project title: Bovine and Equine Bleeding, UI IACUC # 2020-42).

## Results and discussion

### ELQ-300 and ELQ-316 inhibit the growth of *B. bovis, B. bigemina*,* B. caballi* and *T. equi*

**Fig. 1:**
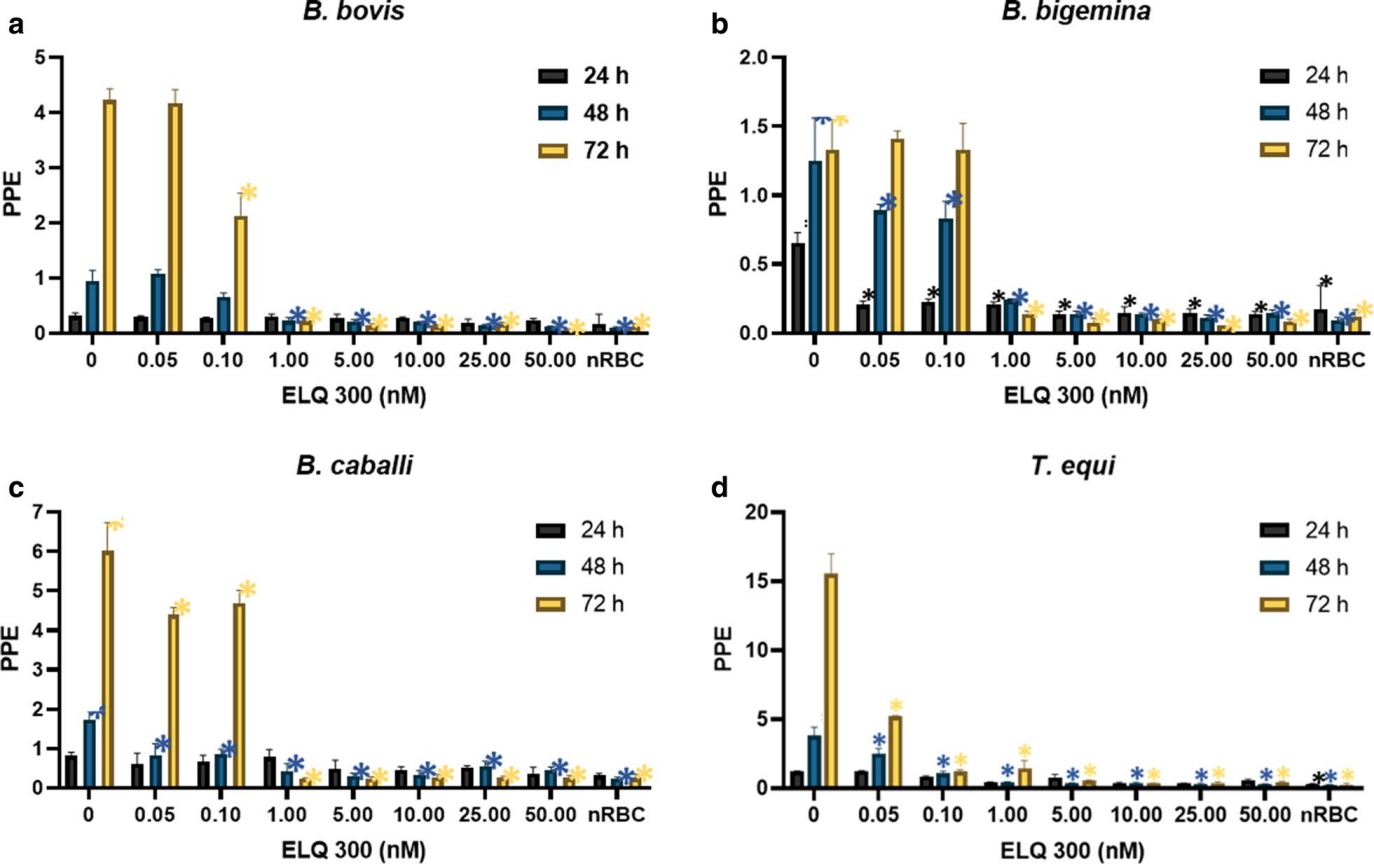
Parasite culture growth at 24 h (purple bars), 48 h (blue bars) and 72 h (yellow bars) without and after addition of different concentrations of endochin-like quinolone-300 (*ELQ-300*). **a** *Babesia bovis*, **b** *B. bigemina* **c** *B. caballi*, **d** *Theilera equi*. “0” represents parasites in the absence of ELQ-300. Assays were carried out in triplicate, and the error bars indicate standard error deviation for each ELQ-300 concentration tested. Asterisk (*) represents statistically significant difference between cultures grown without and with ELQ-300 at *P* < 0.05, using Student’s t-test.* nRBC* Non-infected erythrocytes,* PPE* percentage of parasitized erythrocytes

**Fig. 2:**
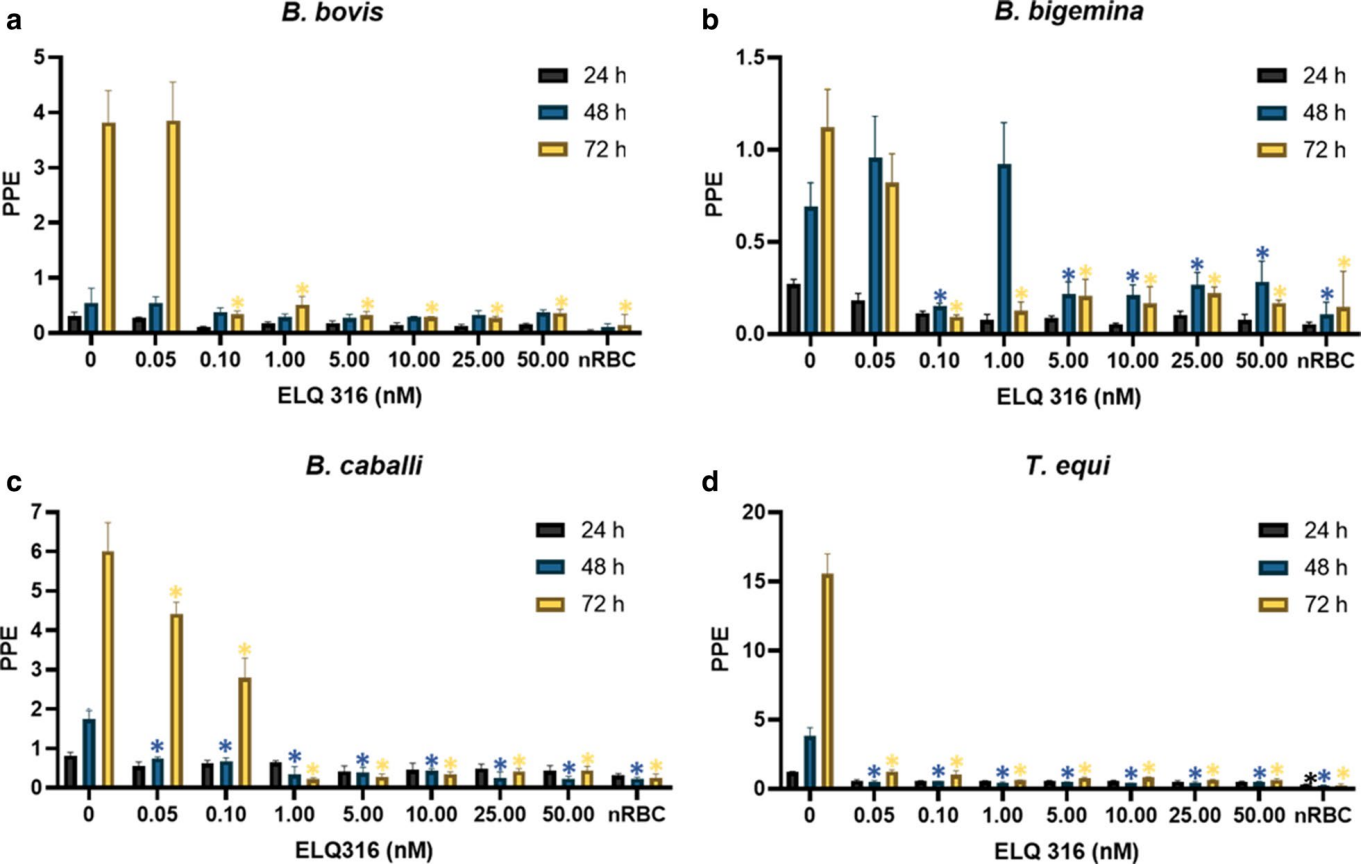
Parasite culture growth at 24 h (purple bars), 48 h (blue bars) and 72 h (yellow bars) without and after addition of different concentrations of ELQ-316. **a** *B. bovis*, **b** *B. bigemina*, **c** *B. caballi*, **d** *T. equi*. “0” represents parasites in the absence of ELQ-316. Assays were carried out in triplicate, and the error bars indicate standard error deviation for each ELQ-316 concentration tested. Asterisk (*) represents statistically significant difference between cultures grown without and with ELQ-316 at *P* < 0.05, using Student’s t-test

The effect of ELQ-300 and ELQ-316 on parasite growth, with a starting PPE of 0.2%, was evaluated using seven different concentrations of each compound, ranging from 0.05 to 50 nM. Both tested drugs significantly inhibited (*P* < 0.05) the growth of *B. bovis*,* B. bigemina*,* B. caballi* and *T. equi* (Figs. 1a–d, 2a–d). In addition, the inhibitory effect of ELQ-300 and ELQ-316 was found to be dose-dependent for all four parasites tested. The calculated IC_50_ and IC_100_ values of ELQ-300 and ELQ-316 for each parasite are shown in Table 1. Overall, comparisons of the IC_50_ values among all parasites tested indicate increased susceptibility to ELQ-316 compared to ELQ-300. The ELQ-316 IC_50_ varied from 0.002 to 0.1 nM, while the ELQ-300 IC_50_ varied from 0.04 to 0.37 nM, as measured at 72 h of culture (Table 1).

**Table 1:** Half maximal and maximal inhibitory concentrations of endochin-like quinolone (ELQ)-300 and ELQ-316 calculated for the four studied species of apicomplexan parasites at 72 h of culture

Species	ELQ-300		ELQ-16	
	IC_50_ (nM)	IC_100_ (nM)	IC_50_ (nM)	IC_100_ (nM)
*Babesia bovis*	0.09 ± 0.002	4.2 ± 0.10	0.07	3.8 ± 0.12
*B. bigemina*	0.37 ± 0.19	1.3 ± 0.05	0.05	3.0 ± 0.15
*B. caballi*	0.19 ± 0.04	5.7 ± 0.24	0.1 ± 0.006	6.0 ± 0.18
*Theilera equi*	0.04 ± 0.003	3.36 ± 0.83	0.002	1.0 ± 1.55

Values in table are presented as the mean and standard deviation (SD) based on triplicates for each experiment.

IC_50_ and IC_100_, half maximal and maximal inhibitory concentrations, respectively

Interestingly, our calculated values of IC_50_ for ELQ-300 and ELQ-316 are in the same range or lower than values estimated for other related apicomplexans in previous studies. ELQ-316 IC_50_ values of 7.97, 0.66 and 0.35 nM were established for *Besnoitia besnoiti *and *Toxoplasma gondii* tachyzoites, respectively [28, 29]. In addition, a previous study demonstrated ELQ-300 IC_50_ values of 15.4 and 23.1 nM for *Plasmodium knowlsei* and *P. falciparum*, respectively [15]. In addition to the acceptable IC_50_ inhibitory values found for ELQ-300, our study showed even lower ELQ-316 IC_50_ values for *B. bovis*, *B. bigemina*, *B. caballi* and *T. equi*, suggesting that these parasites are also highly susceptible to these two drugs. The IC_50_ values obtained for ELQ-300 and ELQ-316 are also lower than those shown with anti-babesial drugs in recently published studies, but in the same IC_50_ range of imidocarb dipropionate for *B. bovis* and *B. bigemina* (Additional file 2: Table S1).

ELQ-300 and ELQ-316 consistently completely abrogated the growth of all four parasites when tested at their respective IC_100_. The calculated IC_100_ values ranged from 1.3 to 5.7 nM for ELQ-300, and from 1.0 to 6.0 nM for ELQ-316 (Table 1). Overall, *B. bigemina*, displayed the lowest IC_100_ value of the four parasites tested and thus appears to be the most susceptible parasite to ELQ-300. On the other hand and based on the IC_100_ values (Table 1), *T. equi* appears to be more susceptible to ELQ-316 than the other four parasites tested in this study. Taking the IC_50_ and IC_100_ data together, we conclude that ELQ-300 and ELQ-316 are able to efficiently inhibit the *in vitro* growth of *B. bovis*, *B. bigemina*, *B. caballi* and *T. equi* blood stages. Notably, while the calculated IC_100_ of *T. equi* is unexpectedly high (500-fold higher than the IC_50_) (Table 1), we cannot rule out the possibility that the actual concentration of the drug in the culture well was affected by poor solubility in the culture media.

### Growth inhibitory effect of ELQ-300 and ELQ-316 is independent of initial parasitemia

**Fig. 3:**
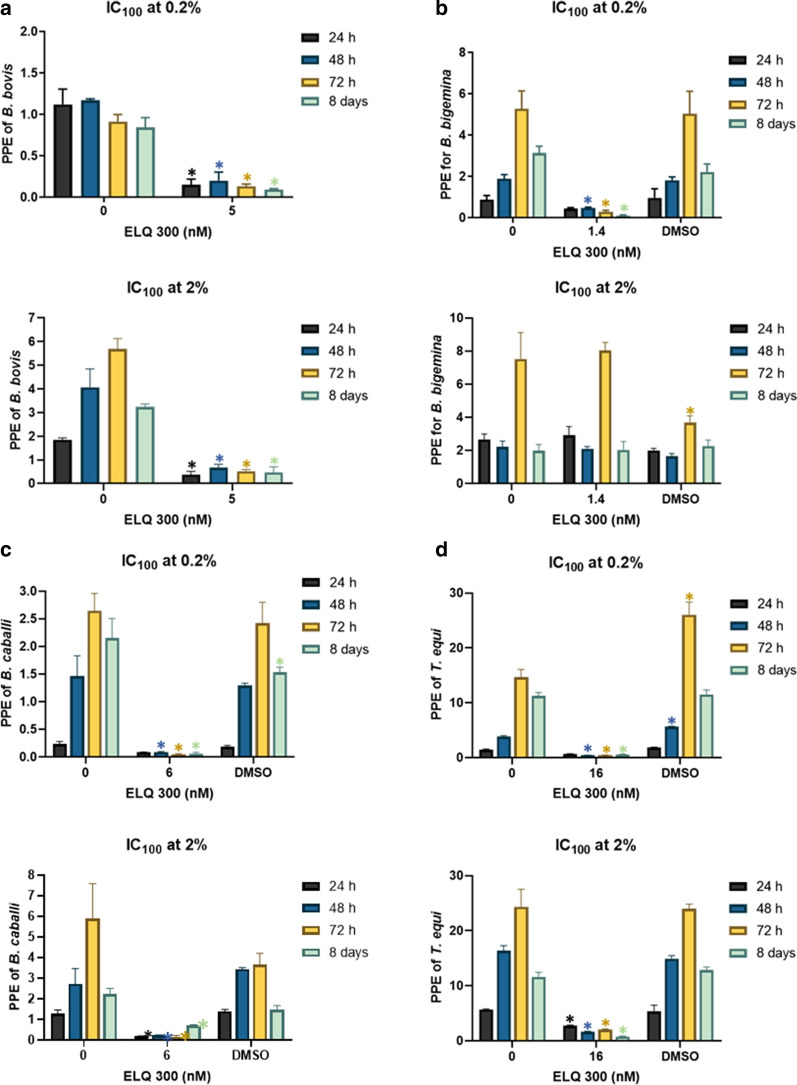
Parasite culture growth at 24 h (purple bars), 48 h (blue bars), and 72 h (yellow bars) using IC_100_ of ELQ-300, and at 8 days (green bars). **a** *B. bovis*, **b** *B. bigemina*, **c** *B. caballi*, **d** *T. equi*. Upper panel of each figure part represents 0.2% PPE, lower panel 2% PPE. “0” represents parasites grown without addition of ELQ-300. Assays were carried out in triplicate and the error bars indicate standard error deviation. Asterisk (*) represents statistically significant difference between cultures grown without and with ELQ-300 at *P* < 0.05, using Student’s t-test.* C*_*50*_ and* IC*_*100*_ Half maximal and maximal inhibitory concentrations, respectively,* DMSO* dimethyl sulfoxide

We then tested whether the efficiency of the compounds is dependent on the parasite initial parasitemia, by comparing the effects of ELQ-300 and ELQ-316, at their respective IC_100_, on the four parasites growing in* in vitro* cultures with starting PPEs of 0.2 and 2%. Neither* B. bovis*,* B. caballi* nor *T. equi* were able to grow in* in vitro* cultures in the presence of the ELQ-300 IC_100_, regardless of their initial PPE (*P* < 0.05) (Fig. 3a, c, d). Nonetheless, the addition of ELQ-300 to *B. bigemina* cultures at an initial PPE of 2% did not result in a rapid decrease of parasitemia (Fig. 3b), in contrast to what was found when the initial PPE was 0.2% (Fig. 3b).

Based on these results, a parasite rescue experiment was performed in which the parasites were grown in culture in the presence of ELQ-300 for 3 days, following which the cultures were split 1:10 and maintained in media free of the drug for an additional 5 days of culture. Parasite growth was not detected (*P* < 0.05) by the end of this period of time for *B. bovis* and *T. equi*, but that was not the case for *B. bigemina* and *B. caballi* (Fig. 3b, c). These results suggest the absence of pre-existing ELQ-300-resistant parasite subpopulations in the *B. bovis* and *T. equi* strains with the ability to survive the initial drug-inhibitory treatment among the parasite strains tested. Collectively, these results are consistent with the relatively increased tolerance of *B. bigemina* and *B. caballi* to ELQ-300, compared to the other two parasites tested, as shown in Fig. 1b and c.

**Fig. 4:**
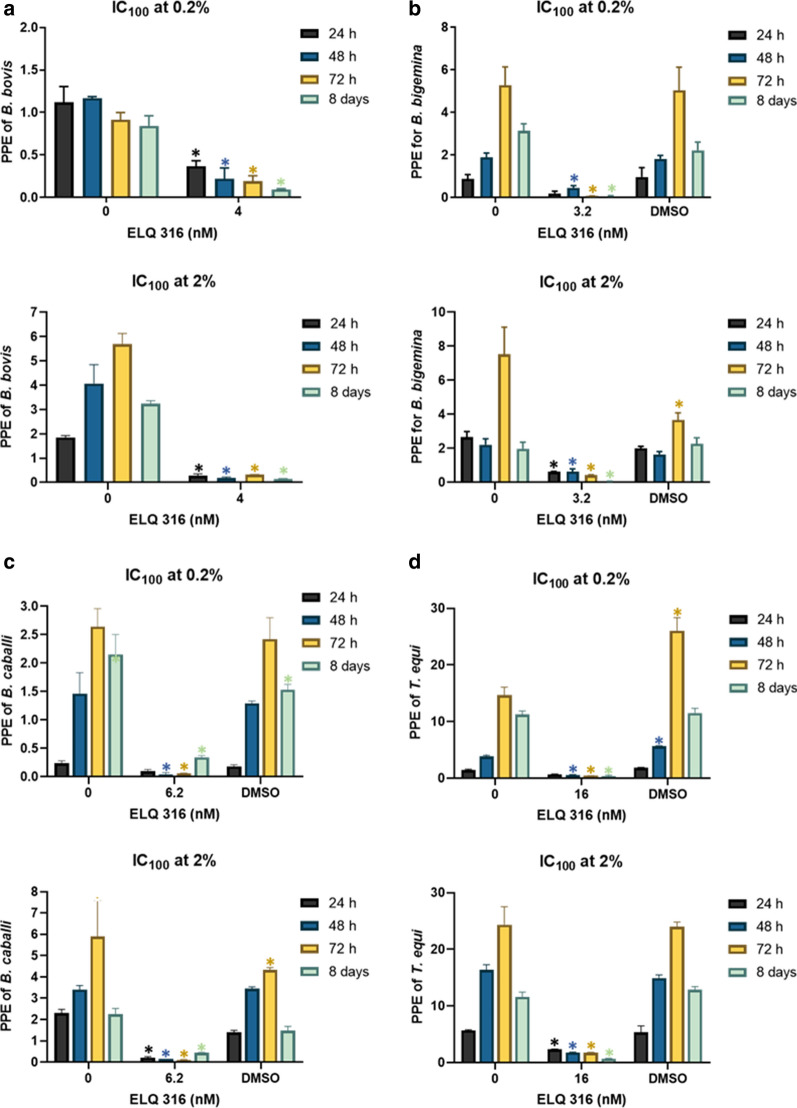
Parasite culture growth at 24 h (purple bars), 48 h (blue bars), and 72 h (yellow bars) using the IC_100_ of ELQ-316, and at 8 days (green bars). **a** *B. bovis*, **b** *B. bigemina*, **c** *B. caballi*, **d** *T. equi*. Upper panel of each figure part represents 0.2% PPE and lower panel represents 2% PPE. “0” represents parasites grown without the addition of ELQ-316. Assays were carried out in triplicate and the error bars indicate standard error deviation. Asterisk (*) represents statistically significant difference between cultures grown without and with ELQ-300 at *P* < 0.05, using Student’s t-test

Interestingly, none of the four species of parasites tested in this study was able to grow in the presence of the ELQ-316 IC_100_ regardless of their initial PPE at 72 h (*P* < 0.05) (Fig. 4a–d). The same lack of parasite growth was observed after 8 days in the parasite rescue experiment, with the exception of *B. caballi* (Fig. 4a–d), independent of the starting PPE. A possible interpretation of these results is that the *B. caballi* strain used in this study may contain a mix of subpopulations of parasites, each one with distinct degrees of tolerance for ELQ-316. In contrast, the *B. bovis*, *B. bigemina* and *T. equi* strains used in these experiments appear to be composed of subpopulations that are highly susceptible to ELQ-316. It was beyond the scope of this study to investigate the mechanism involved in the susceptibility to the ELQ drugs. However, it may be speculated that such susceptibility can be due to variations/mutations in the cytochrome* bc*_1_ target sequence that affect ELQ binding, differential uptake or elimination of the drugs or a combination of these factors (Additional file 3: Figure S2) [29–31]. It was recently shown that genetic alterations in the Q_i_ binding site of the cytochrome* bc*_1_ complex (*Cytb*) of *B. microti* is associated with resistance to ELQ-316, which suggests that this cytochrome gene is as a potential target for the ELQ drugs [16]. Based on these observations, we performed alignment analysis of the *Cytb* genes of *B. bovis*, *B. bigemina*, *B. caballi* and *T. equi* together with the *B. microti Cytb*. Our results indicated full conservation of the two canonical Q_o_ and Q_i_ binding sites of *Cytb* in all sequences analyzed and a high level of amino acid identity, which ranged from 47.2 to 49.6 % in comparison to *B. microti* (Fig. 2) (Additional file 2: Table S2). Overall, the results presented here set the rationale for further studies to alter and/or knockdown the *Cytb* gene in these parasites and evaluate its potential effect on the susceptibility or resistance to the ELQ drugs.

### ELQ-300 and ELQ-316 do not affect viability of equine and bovine PBMC

**Fig. 5:**
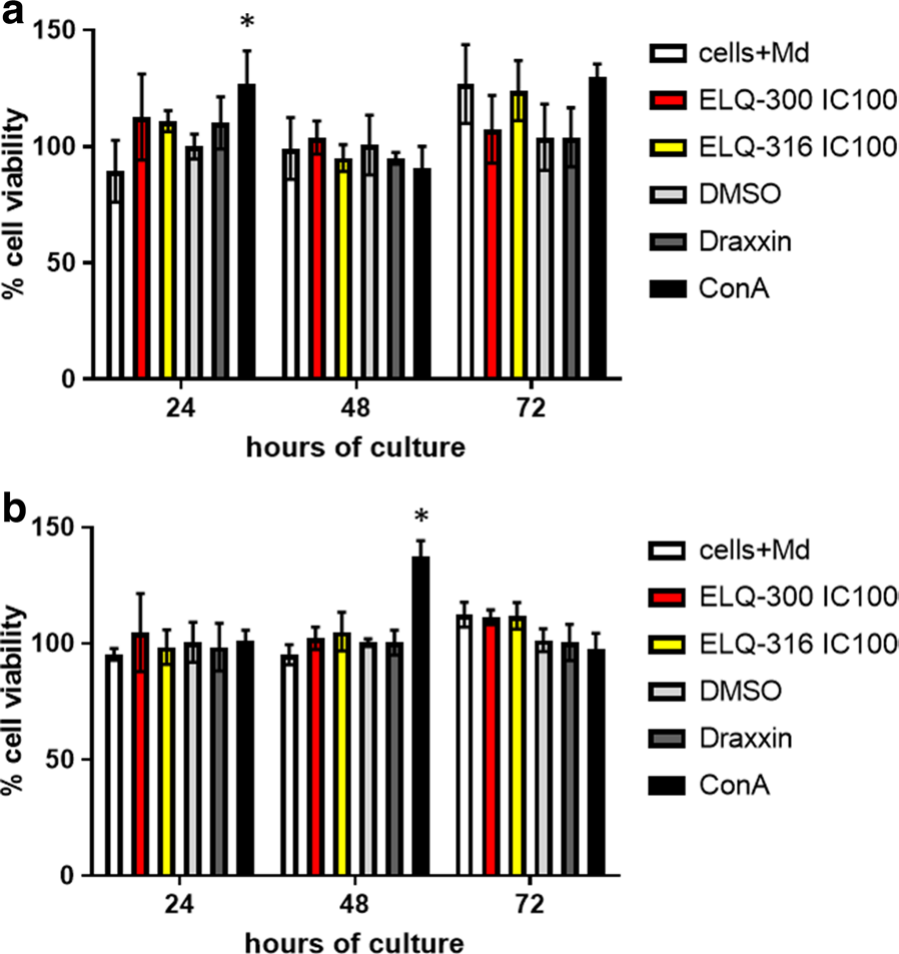
Percentage of cell viability over a period of 72 h after incubation with the IC_50_ and IC_100_of ELQ-300 and ELQ-316.* Cells+Med* Peripheral blood mononuclear cells (PBMC) cultivated without the addition of the ELQ compounds. Cells in cultured in DMSO and Draxxin® were used as a negative control; concanavalin A (*ConA*) was used as a positive control for cell proliferation. **a** Bovine PBMC, **b** horse PBMC. Bovine and horse PBMC assays were carried out in triplicate and the error bars indicate standard error deviation. Asterisk (*) represents statistically significant differences compared to PBMC cultivated in medium only at *P* < 0.05, using Student’s t-test

Cytotoxic assays were performed to assess whether ELQ-300 and ELQ-316 affect the viability of equine and bovine PBMC, which we used as surrogates of nucleated vertebrate host cells. The cytotoxic assays were performed using the IC_100_ doses of ELQ-300 and ELQ-316 in* in vitro* cultures. For the bovine PBMC experiment, the ELQ-300 IC_100_ of 4.3 nM and ELQ-316 IC_100_ of 3.92 nM were used, respectively, whereas for the equine PBMC experiment, the ELQ-300 IC_100_ of 5.94 nM and ELQ-316 IC_100_ of 6.18 nM were used, respectively. Viability of PBMC was similar regardless of the presence or absence of parasite lethal doses of ELQ-300 or ELQ-316, strongly suggesting that cell viability was not compromised by any of these two drugs under the experimental conditions used in the assays (Fig. 5a, b). In addition, a significant increase (*P* <0.05) in cell proliferation was observed in bovine and horse PBMC exposed to ConA for 24 and 48 h, respectively (Fig. 5a, b), indicating adequate sensitivity for the WST-1 proliferation assay used in this study. Taken together, results of the cell viability study revealed that ELQ-300 and ELQ-316, at their respective IC_100_, lack a significant toxic effect on bovine and horse PBMC cultured* in vitro*. Although the data presented here strongly suggest that these two drugs are appropriate candidates for the treatment of BB and EP, it needs to be pointed out that we did not assess their effect* in vivo* and that our evidence on the effectivity and safety of ELQ-300 and ELQ-316 was obtained from testing the effect of the drugs on parasites growing in culture. In addition, investigation of the mechanism of action of ELQ-300 and ELQ-316 in the parasites studied herein was also beyond the scope of this study, and this aspect needs further examination.

## Conclusions

Overall, the results presented here demonstrate that both of the drugs tested in this study, ELQ-300 and ELQ-316, are efficient in inhibiting the growth of* in vitro*-cultured *B. bovis*, *B. bigemina*, *B. caballi* and *T. equi*. Importantly, the IC_100_ doses of the ELQ drugs did not significantly affect the viability of* in vitro*-cultured cattle and horse PBMC. Collectively, the findings of this study strongly suggest that ELQ-300 and ELQ-316 can be potentially effective and safe candidates for the development of novel therapies to control BB and EP. However, it will be important to confirm their mechanisms of action, as well as the drugs’s potential to select for resistant strains. Further studies* in vivo* in horses and bovines are needed to evaluate the efficacy of ELQ-300 and ELQ-316 against acute and chronic BB and EP.

## Supplementary information

**Additional file 1:Fig. S1.** Chemical structures of ELQ-300 (**a**) and ELQ-316 (**b**).

**Additional file 2:Table S1. ** IC_50_ calculated for different compounds tested against* in vitro* in *B. bovis*, *B. bigemina*, *B. caballi* and *T. equi* cultures. **Table S2.** Amino acid percentage identity of cytochrome* bc*1 complex (*Cytb*) of *Babesia bovis*, *B. bigemina*, *B. caballi*, and *Theileria equi* in comparison to *B. microti*.

**Additional file 3: Fig. S2.** Alignment of amino acid sequences of the cytochrome* bc*1 complex (*Cytb*) of* B. bovis* (GenBank accession YP_001504108), *B. bigemina* (GenBank accession BAI66164.1), *B. caballi* (GenBank accession BAI66167.1), * T. equi* (GenBank accession XP_025033545.1), and *B. microti* (GenBank accession MT114078).

## Data Availability

The datasets supporting the conclusions of this article are included within the article and its Additional files 1, 2 and 3.

## Abbreviations

Con A: Concanavalin A
DMSO: Dimethyl sulfoxide
ELQ: Endochin-like quinolones
HE: Hydroethidine
IC: Inhibitory concentration
PBMC: Peripheral blood mononuclear cells
PBS: Phosphate buffer saline
PPE: Percentage of parasitized erythrocytes
