# Supplementary figures and images for: Endochin-like quinolone-300 and ELQ-316 inhibit Babesia bovis, B. bigemina, B. caballi and Theileria equi
Source: Parasit Vectors. 2020 Dec 3;13:606. doi: 10.1186/s13071-020-04487-3 (PMC7712603; doi:10.1186/s13071-020-04487-3)

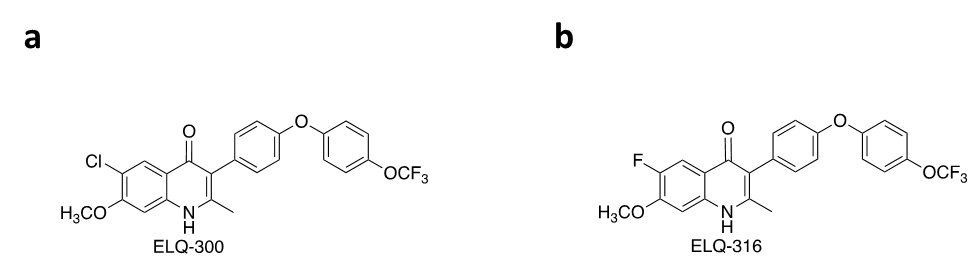

Supplement: Supplementary file 1 — Additional file 1:Fig. S1. Chemical structures of ELQ-300 (a) and ELQ-316 (b). [file 13071_2020_4487_MOESM1_ESM.tiff]

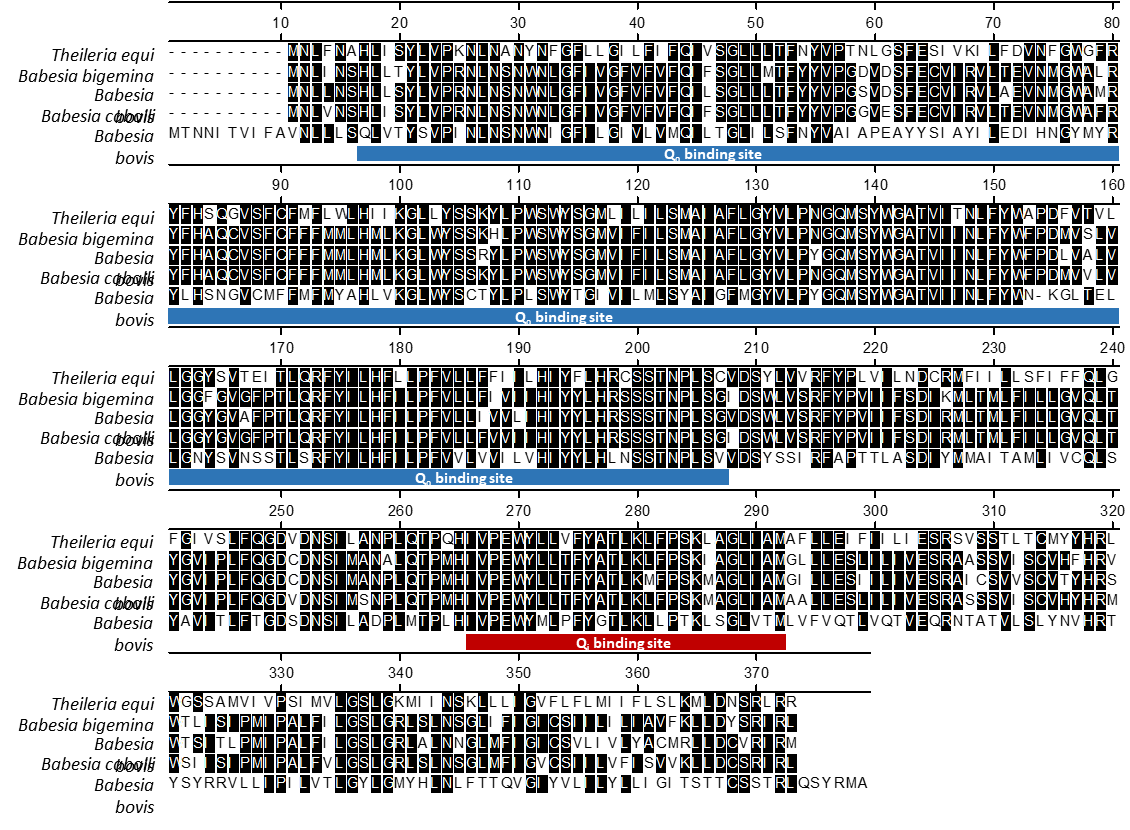

Supplement: Supplementary file 3 — Additional file 3: Fig. S2. Alignment of amino acid sequences of the cytochrome bc1 complex (Cytb) of B. bovis (GenBank accession YP_001504108), B. bigemina (GenBank accession BAI66164.1), B. caballi (GenBank accession BAI66167.1), T. equi (GenBank accession XP_025033545.1), and B. microti (GenBank accession MT114078). [file 13071_2020_4487_MOESM3_ESM.tiff]
