# Supplementary material for: Endochin-like quinolone-300 and ELQ-316 inhibit Babesia bovis, B. bigemina, B. caballi and Theileria equi
Source: Parasit Vectors. 2020 Dec 3;13:606. doi: 10.1186/s13071-020-04487-3 (PMC7712603; doi:10.1186/s13071-020-04487-3)
Supplement: Supplementary file 2 — Additional file 2:Table S1. IC50 calculated for different compounds tested against in vitro in B. bovis, B. bigemina, B. caballi and T. equi cultures. Table S2. Amino acid percentage identity of cytochrome bc1 complex (Cytb) of Babesia bovis, B. bigemina, B. caballi, and Theileria equi in comparison to B. microti. [file 13071_2020_4487_MOESM2_ESM.docx]

**Table S1** IC50 calculated for different compounds tested against in vitro *B. bovis*, *B. bigemina*, *B. caballi* and *T. equi* cultures.

| **Drug compound [Ref.]** | **Parasite Species** | **IC_50_** |
| --- | --- | --- |
| Atovaquone (AV) [1, 2] | *B. bovis*  *B. bigemina*  *B. caballi*  *T. equi* | 39.7 ± 2.4 nM *, 2.38 ± 0.53 nM  706.1 ± 38.7 nM  101.9 ± 14.1 nM  95.0 ± 65 nM |
| Camptothecin [3] | *B. bovis*  *B. bigemina*  *B. caballi*  *T. equi* | 11.67 ± 1.6 μM  4.00 ± 1.0 μM  2.07 ± 0.6 μM  0.33 ± 0.02 μM |
| Chalcone 4 hydrate (CH) [4] | *B. bovis*  *B. bigemina*  *B. caballi*  *T. equi* | 138.4 ± 4.4 µM  60.9 ± 1.1 µM  27.9 ± 1.2 µM  19.2 ± 1.5 μM |
| Trans-chalcone (TC) [4] | *B. bovis*  *B. bigemina*  *B. caballi*  *T. equi* | 69.6 ± 2.3 µM  33.3 ± 1.2 µM  18.9 ± 1.7 µM  14.3 ± 1.6 μM |
| Clofazimine  Tuvshintulga et al., 2016 | *B. bovis*  *B. bigemina*  *B. caballi*  *T. equi* | 4.5 ± 0.3 μM  3.0 ± 0.2 μM  4.3 μM  0.29 ± 0.03 μM |
| Cryptolepine hydrate (CRY) [5] | *B. bovis*  *B. bigemina*  *B. caballi*  *T. equi* | 1740 ± 0.377  1400 ± 0.6 µM  600 ± 0.53 µM  730 ± 0.025 nM |
| 17-dimethylaminoethylamino-17-demethoxygeldanamycin (17-DMAG) [1] | *B. bovis*  *B. bigemina*  *B. caballi*  *T. equi* | 77.6 ± 2.9 nM  62.4 ± 1.9 nM  88.5 ± 9.6 nM  307.7 ± 7 nM |
| Diminazene aceturate [1, 6] | *B. bovis*  *B. bigemina*  *B. caballi*  *T. equi* | 189.8 ± 42.1 nM , 0.48 ± 0.09 μM  1852 ± 104 nM , 0.21 ± 0.06 μM  13.4 ± 3.6 nM , 0.005 ± 0.0002 μM  59.9 ± 8.5 nM |
| Draxxin® (Tulathromycin) [7] | *B. bovis*  *B. bigemina*  *T. equi* | 16.7 ± 0.6 nM  6.2 ± 0.2 nM  2.4 ± 0.1 nM |
| Ellagic acid (EA) [8] | *B. bovis*  *B. bigémina*  *B. caballi*  *T. equi* | 9.58 ± 1.47 µM  7.87 ± 5.8 µM  3.29 ± 0.42 µM  7.46 ± 0.6 µM |
| β-cyclodextrin ellagic acid (β-CD EA) [8] | *B. bovis*  *B. bigémina*  *B. caballi*  *T. equi* | 8.8 ± 0.53 µM  18.9 ± 0.025 µM  7, 4.4 ± 0.6 µM  9.1 ± 1.72 µM |
| Antisolvent precipitation with a syringe pump prepared ellagic acid (APSP EA) [8] | *B. bovis*  *B. bigémina*  *B. caballi*  *T. equi* | 4.2 ± 0.42 µM  9.6 ± 0.6 µM  0.92 ± 5.8 µM  7.3 ± 0.54 µM |
| Imidocarb dipropionate [7, 9, 10] | *B. bovis*  *B. bigemina*  *T. equi* | 8.6 nM  0.08 nM  279 nM |
| N-acetyl-L-cysteine [6] | *B. bovis*  *B. bigemina*  *B. caballi*  *T. equi* | 332.1 ± 33.1 μM  229.2 ± 37.5 μM  114.5 ± 28.6 μM  349.1 ± 16.2 μM |
| Trifluralin analogues (TFLA) 2, TFLA 11, TFLA 13 and TFLA [11] | *B. bovis*  *B. bigemina*  *B. caballi*  *T. equi* | 13.1±3.0, 12.1±1.1, 8.9±0.7, 13.4±1.5 μM  10.0±0.6, 8.2±1.0, 9.0±1.1, 9.3±1.7 μM  6.5±0.5, 5.1±0.2, 5.1±0.1, 5.5±0.2 μM  7.7±0.3, 7.6±0.4, 6.3±0.1, 5.8±0.2 μM |

**References (Table S1)**

1. Guswanto A, Nugraha AB, Tuvshintulga B, Tayebwa DS, Rizk MA, Batiha GE, Gantuya S, Sivakumar T, Yokoyama N, Igarashi I. 17-DMAG inhibits the multiplication of several *Babesia* species and *Theileria equi* on *in vitro* cultures, and *Babesia microti* in mice. Int J Parasitol Drugs and Drug Resistance 2018, 8(1):104-111.

2. Kamyingkird K, Cao S, Tuvshintulga B, Salama A, Mousa AA, Efstratiou A, Nishikawa Y, Yokoyama N, Igarashi I, Xuan X. Effects of dihydroorotate dehydrogenase (DHODH) inhibitors on the growth of Theileria equi and Babesia caballi in vitro. Exp Parasitol 2017, 176:59-65.

3. Tayebwa DS, Tuvshintulga B, Guswanto A, Nugraha AB, Batiha GE, Gantuya S, Rizk MA, Vudriko P, Sivakumar T, Yokoyama N *et al*. The effects of nitidine chloride and camptothecin on the growth of *Babesia* and *Theileria* parasites. Ticks and Tick Borne Dis 2018, 9(5):1192-1201.

4. Batiha GE, Beshbishy AM, Tayebwa DS, Adeyemi OS, Shaheen H, Yokoyama N, Igarashi I. The effects of trans-chalcone and chalcone 4 hydrate on the growth of *Babesia* and *Theileria*. PLoS Negl Trop Dis 2019, 13(5):e0007030.

5. Batiha GE, Beshbishy AM, Alkazmi LM, Nadwa EH, Rashwan EK, Yokoyama N, Igarashi I. *In vitro* and *in vivo* growth inhibitory activities of cryptolepine hydrate against several *Babesia* species and *Theileria equi*. PLoS Neg Trop Dis 2020, 14(8):e0008489.

6. Rizk MA, El-Sayed SAE, AbouLaila M, Yokoyama N, Igarashi I: Evaluation of the inhibitory effect of N-acetyl-L-cysteine on *Babesia* and *Theileria* parasites. Exp Parasitol 2017, 179:43-48.

7. Silva MG, Villarino NF, Knowles DP, Suarez CE. Assessment of Draxxin((R)) (tulathromycin) as an inhibitor of *in vitro* growth of *Babesia bovis*, *Babesia bigemina* and *Theileria equi*. Int J Parasitol Drugs and Drug Resistance 2018, 8(2):265-270.

8. Beshbishy AM, Batiha GE, Yokoyama N, Igarashi I. Ellagic acid microspheres restrict the growth of *Babesia* and *Theileria in vitro* and *Babesia microti in vivo*. Parasite Vector 2019, 12(1):269.

9. Nott SE, O'Sullivan WJ, Gero AM, Bagnara AS. Routine screening for potential babesicides using cultures of *Babesia bovis*. Int J Parasitol 1990, 20(6):797-802.

10. Gopalakrishnan A, Maji C, Dahiya RK, Suthar A, Kumar R, Gupta AK, Dimri U, Kumar S. *In vitro* growth inhibitory efficacy of some target specific novel drug molecules against *Theileria equi*. Vet Parasitol 2016, 217:1-6.

11. Silva MG, Domingos A, Esteves MA, Cruz ME, Suarez CE. Evaluation of the growth-inhibitory effect of trifluralin analogues on *in vitro* cultured *Babesia bovis* parasites. Int J Parasitol Drugs and Drug Resistance 2013, 3:59-68.

**Table S2** Amino acid percent identity of cytochrome bc1 complex (*Cytb*) of *Babesia bovis*, *B. bigemina*, *B. caballi*, and *Theileria equi* in comparison to *B. microti*.

|  | *B. bovis* | *B. bigemina* | *B. caballi* | *T. equi* |
| --- | --- | --- | --- | --- |
| *B. microti* | 49.6% | 48.5% | 49.6% | 47.2% |
